# Physiological tonicity improves human chondrogenic marker expression through nuclear factor of activated T-cells 5 *in vitro*

**DOI:** 10.1186/ar3031

**Published:** 2010-05-21

**Authors:** Anna E van der Windt, Esther Haak, Ruud HJ Das, Nicole Kops, Tim JM Welting, Marjolein MJ Caron, Niek P van Til, Jan AN Verhaar, Harrie Weinans, Holger Jahr

**Affiliations:** 1 Department of Orthopaedics, Erasmus MC, University Medical Center Rotterdam, Dr. Molewaterplein 50, 3015 GE Rotterdam, The Netherlands; 2 Department of Orthopaedic Surgery, GROW school for Oncology and Developmental Biology, Maastricht University Medical Center, Universiteitssingel 40, 6202 AZ Maastricht, The Netherlands; 3 Department of Hematology, Erasmus MC, University Medical Center Rotterdam, Dr. Molewaterplein 50, 3015 GE Rotterdam, The Netherlands

## Abstract

**Introduction:**

Chondrocytes experience a hypertonic environment compared with plasma (280 mOsm) due to the high fixed negative charge density of cartilage. Standard isolation of chondrocytes removes their hypertonic matrix, exposing them to nonphysiological conditions. During *in vitro *expansion, chondrocytes quickly lose their specialized phenotype, making them inappropriate for cell-based regenerative strategies. We aimed to elucidate the effects of tonicity during isolation and *in vitro *expansion on chondrocyte phenotype.

**Methods:**

Human articular chondrocytes were isolated and subsequently expanded at control tonicity (280 mOsm) or at moderately elevated, physiological tonicity (380 mOsm). The effects of physiological tonicity on chondrocyte proliferation and chondrogenic marker expression were evaluated. The role of Tonicity-responsive Enhancer Binding Protein in response to physiological tonicity was investigated using nuclear factor of activated T-cells 5 (NFAT5) RNA interference.

**Results:**

Moderately elevated, physiological tonicity (380 mOsm) did not affect chondrocyte proliferation, while higher tonicities inhibited proliferation and diminished cell viability. Physiological tonicity improved expression of chondrogenic markers and NFAT5 and its target genes, while suppressing dedifferentiation marker collagen type I and improving type II/type I expression ratios >100-fold. Effects of physiological tonicity were similar in osteoarthritic and normal (nonosteoarthritic) chondrocytes, indicating a disease-independent mechanism. NFAT5 RNA interference abolished tonicity-mediated effects and revealed that NFAT5 positively regulates collagen type II expression, while suppressing type I.

**Conclusions:**

Physiological tonicity provides a simple, yet effective, means to improve phenotypical characteristics during cytokine-free isolation and *in vitro *expansion of human articular chondrocytes. Our findings will lead to the development of improved cell-based repair strategies for chondral lesions and provides important insights into mechanisms underlying osteoarthritic progression.

## Introduction

Hyaline articular cartilage is a connective tissue covering the ends of bones in joints and is composed of specialized cells, chondrocytes that produce a large amount of extracellular matrix. This matrix is crucial for the unique biomechanical properties of this tissue and is composed of a collagen fiber network, providing tensile strength and flexibility, and abundant ground matrix rich in proteoglycans [1].

The glycosaminoglycan (GAG) side chains of the proteoglycans are sulfated and responsible for a characteristic high fixed negative charge density [2], which binds mobile cations (mainly sodium). This binding determines the physiological tonicity (that is, osmotic pressure) of the extracellular fluid around chondrocytes *in vivo*, but the tonicity indirectly also largely depends on the quality of the collagen network. Extracellular tonicity in healthy cartilage ranges between 350 and 480 mOsm [3,4]. *In vivo*, tonicity of the extracellular fluid is dynamic and changes due to alterations in matrix hydration [5]. During cartilage degeneration (that is, in osteoarthritis (OA)), the collagen matrix degrades and the GAG concentration diminishes, resulting in a severity-dependent decreased tonicity of between 280 and 350 mOsm [3,6]. Currently, chondrocyte isolation and *in vitro *expansion culture are performed in medium of nonphysiological tonicity (270 ± 20 mOsm). Several studies have already shown that chondrocytes are tonicity responsive [7-9] and react with changes in matrix synthesis [4,8,10,11], but focused on aggrecan (AGC1) core protein mRNA levels, AGC1 promoter activity and GAG production.

Molecular mechanisms involved in the hypertonic response of human articular chondrocytes (HACs) are poorly understood. Hypertonicity perturbs cells by causing osmotic efflux of water, resulting in cell shrinkage [12,13]. Cells react by a rapid uptake of ions, which increase cellular ionic strength [14] with potentially detrimental effects [15-17]. The initial, rapid response is the activation of transporters that exchange these ions for compatible osmolytes [16,18]. This process is controlled by Tonicity-responsive Enhancer Binding Protein (TonEBP/NFAT5), which mediates transcriptional activation of these transporters [16]. Nuclear factor of activated T-cells 5 (NFAT5) is a member of the Rel family of transcription factors [19] and targets sodium/myo-inositol cotransporter (SMIT) [20,21], sodium/chloride-coupled acid transporters (BGT1/SLC6A12) [20], aquaporin channels (AQP1 and AQP2) [22], and calcium-binding proteins (S100A4) [23-25]. Upon hypertonic stress, transcription of NFAT5 itself is upregulated in several cell types [26-28], but the tonicity threshold and cell signaling pathways required to activate NFAT5 may be cell type specific [29]. Nothing is currently known about the expression or function of NFAT5 in HACs.

Chondral lesions from, for example, trauma or overuse, can cause joint pain, immobility and eventually OA. The associated high prevalence - 60% of all patients undergoing knee arthroscopy are diagnosed with a chondral lesion [30] - and loss of quality of life makes cartilage damage a major personal and economical burden. Treatment options for chondral lesions are limited, and autologous chondrocyte implantation is the currently most developed hyaline repair technique for the knee [31]. Characterized chondrocyte implantation, employing phenotypical prescreening prior to implantation, has recently improved structural repair [32].

Chondrocyte dedifferentiation during *in vitro *expansion for autologous chondrocyte implantation is detrimental; but almost inevitably in standard monolayer culture, spherical chondrocytes will gradually convert into fibroblast-like cells [33,34]. This morphological change is accompanied by a shift in collagen expression towards less collagen type II (COL2) and more collagen type I (COL1) [34,35]. Consequently, dedifferentiated chondrocytes produce fibrocartilage *in vivo*, with an extracellular matrix of inferior biomechanical properties due to higher collagen (especially type I) content and less proteoglycans compared with native hyaline cartilage [36]. Three-dimensional culture systems can partially prevent dedifferentiation, but are labor intensive and essentially impair propagation. Chondrocyte dedifferentiation might also play a role in the pathogenesis of OA, as the ability of aging chondrocytes to produce and repair the extracellular matrix is compromised [37] and as COL1 is shown to be present in chondrocyte clusters in fibrillated areas of late-stage OA cartilage while it is absent in healthy cartilage [38].

In the present article we report that physiological tonicity (380 mOsm) during isolation and monolayer expansion can suppress chondrocyte dedifferentiation and that expression of the extracelluar matrix components collagen type I and collagen type II as well as aggrecan is NFAT5 dependent. We further show that NFAT5 contributes to the differential regulation of both collagen types. This study provides a simple, yet novel and effective, means to improve cell-based repair strategies for chondral lesions and contribute to our understanding of OA progression.

## Materials and methods

### Cartilage and chondrocyte isolation

After informed consent was obtained, human articular cartilage was explanted from macroscopically normal areas of the femoral condyles and tibial plateau of nine patients undergoing total knee replacement surgery for OA (medical ethical approval MEC2004-322). In addition to preparation of cartilage explants and isolation of HACs under standard conditions (DMEM, 280 mOsm) as described by Das and colleagues [39], medium tonicity was also adjusted to 380 mOsm, 480 mOsm or 580 mOsm by addition of sterile NaCl. Enzymatic digestion, removal of undigested fragments and subsequent chondrocyte culture were all reported earlier [39]. The 280 mOsm and 380 mOsm isolations were also performed with cartilage obtained from the femoral condyles and tibial plateau of two non-OA donors (further referred to as normal donors) undergoing above-knee amputation surgery after trauma.

### Chondrocyte proliferation and DNA measurements

Primary (P0), passage 1 (P1), passage 2 (P2) and passage 3 (P3) HACs were monolayer expanded in medium corresponding to their isolation tonicity (280 mOsm, 380 mOsm, 480 mOsm or 580 mOsm), with an initial seeding density of 6,000 cells/cm^2^. Cells were harvested daily for cell counts and DNA assay between days 2 and 6. Experiments were performed in duplicate from three OA donors (n = 6). At each passage, growth curves were established by cell counts using Trypan Blue (catalogue number T8154; Sigma-Aldrich, St. Louis, MO, USA) and DNA quantification. DNA measurements were performed according to Karsten and Wollenberger [40] with slight modifications [41]. Doubling times within each passage were calculated from the trend line of the exponential growth phase using the equation:

where *k *is the growth constant and *T *is the doubling time.

### Chondrocyte expansion

Primary HACs were cultured for expansion in monolayers at a seeding density of 7,500 cells/cm^2 ^in medium corresponding to their isolation tonicity (280 mOsm, 380 mOsm, 480 mOsm or 580 mOsm). P0 cells to P3 cells were seeded in high-density monolayers (20,000 cells/cm^2^) and were cultured for an additional 5 days and 7 days before analysis of mRNA (quantitative RT-PCR) and protein expression (Western blotting), respectively. Experiments were performed in triplicate from four OA donors (n = 12). Additional experiments were performed in triplicate from two healthy donors (n = 6) to investigate whether the hypertonic stress response is specific for pathologically altered cells. To exclude sodium-specific or chloride-specific effects, we performed experiments using *N*-methyl-d-glucamine chloride (NMDG-Cl) or sucrose to adjust the medium tonicity to 380 mOsm.

### Lentiviral *NFAT5* gene knockdown

We used lentiviral vectors for nontransient shRNA-mediated gene silencing in primary chondrocytes [42]. *Bam*HI/*Mun*I restriction fragments of the parental pLKO.1-puro vector - each containing the U6 promotor and one out of five different, sequence-verified anti-human NFAT5 shDNAs (MISSION shRNA library [43]) - were subcloned into corresponding restriction sites of recipient vector pRRL.PPT.PGK.GFPpre. This vector was kindly provided by L Naldini (San Raffaele Telethon Institute for Gene Therapy, Milan, Italy) [44,45] and was optimized by A Schambach (Department of Experimental Hematology, Hannover Medical School, Hannover, Germany) [46] to express enhanced green fluorescent protein (eGFP) from the phosphoglycerate kinase promoter. Lentiviral particles were produced in HEK293T cells by transient transfection using a calcium phosphate protocol [47]. Cells transduced with a lentiviral vector lacking the NFAT5-specific shRNA expression cassette served as controls. All cells were grown in monolayers. TRCN0000020020 was identified as the best performing anti-NFAT5 shRNA clone by quantitative PCR-based knockdown efficiency determination, and was used in subsequent experiments.

P1 OA HACs from two donors were seeded (15,000 cells/cm^2^) and cultured for 4 days in control medium (280 mOsm). Three hours prior to transduction, cells were deprived of antibiotics, and then were transduced for ± 18 hours, refreshed with control medium with antibiotics and cultured for an additional 4 days before harvesting for fluorescence-activated cell sorting (FACS) analyses. Cells were resuspended in PBS with 10% FCS and antibiotics, and were washed. Cells were collected and stained with Hoechst 33258 (1 mg/ml; Molecular Probes/Invitrogen Corp., Carlsbad, CA, USA) to discriminate between dead cells and live cells. FACS was performed on the FACSAria (Becton Dickinson BV, Breda, The Nederlands), and eGFP-expressing cells were collected (>50%, multiplicity of infection ~1) and reanalyzed for purity (>95%) using Cell Quest Pro Software (Becton Dickinson Biosciences BV, Breda, The Nederlands).

The eGFP-expressing populations were seeded (10,000 cells/cm^2^) and cultured in control medium up to 80% confluency. Cells were then switched to medium of 380 mOsm or were kept on control medium for 24 hours prior to RNA analysis.

### RNA expression analysis

RNA isolation, purification, quantification and cDNA synthesis are described elsewhere [48]. Expression levels of *AGC1*, *SOX9 *and *COL2 *were studied as chondrogenic markers, while *COL1 *was studied as a dedifferentiation marker [34,35,49,50]. Quantitative PCR assays for *COL2*, *SOX9*, *AGC1 *and *COL1 *have been reported earlier [51].

To quantify expression of *NFAT5 *and its target genes, the following primers were tested for similar amplification efficiency and specificity according to Das and colleagues [39], and were used as 20 μl SYBR^® ^Green I reactions: HsNFAT5_Fw, GGGTCAAACGACGAGATTGTG and HsNFAT5_Rv, TTGTCCGTGGTAAGCTGAGAA; HsS100A4_Fw, GTCCACCTTCCACAAGTACTCG and HsS100A4_Rv, TCATCTGTCCTTTTCCCCAAG; and HsSLC6A12_Fw, ACACAGAGCATTGCACGGACT and HsSLC6A12_Rv, CCAGAACTCGTCTCTCCCAGAA. Data were normalized to an index of three reference genes (*GAPDH*, *UBC*, *HPRT1*) that were pre-evaluated to be stably expressed across samples [39]. Relative expression was calculated according to the 2^-ΔCT ^method [52].

### Western blot analysis

Cells seeded at high densities were washed twice with PBS and were lysed in RIPA buffer [53] with addition of protease inhibitors. The total protein concentration was quantified by the bicinchoninic acid assay according to the manufacturer's protocol (#23225; Thermo Fisher Sci., Rockford, IL, USA). Aliquots (10 μg) were subjected to 10% SDS-PAGE prior to electroblotting onto nitrocellulose membranes (Protran BA83; Schleicher & Schuell BV, s-Hertogenbosch, The Netherlands). Blots were blocked in 5% low-fat dry milk in 1× PBS, 0.05% v/v NP-40, were incubated with primary antibodies - anti-type II collagen and anti-type I collagen, both 1:100 (SouthernBiotech, Birmingham, Alabama, USA), or 1:10,000 anti-α-Tubulin (Sigma) - were washed, were incubated with secondary antibodies (both 1:1,000; Dako Cytomation, Heverlee, Belgium) and were chemiluminescently detected. Signals were quantified using ImageJ 1.42 software [54].

### Statistical analysis

Statistical analysis was performed using SPSS 13.0 software (SPSS Inc., Chicago, IL, USA). Data were compared between groups by Kruskall-Wallis H test and *post-hoc *Mann-Whitney U test. Results represent the mean ± standard deviation, and *P *< 0.05, *P *< 0.01 and *P *< 0.001 were considered to indicate levels of statistically significant difference.

## Results

### Hypertonicity influences proliferation and survival of chondrocytes

**Figure 1:**
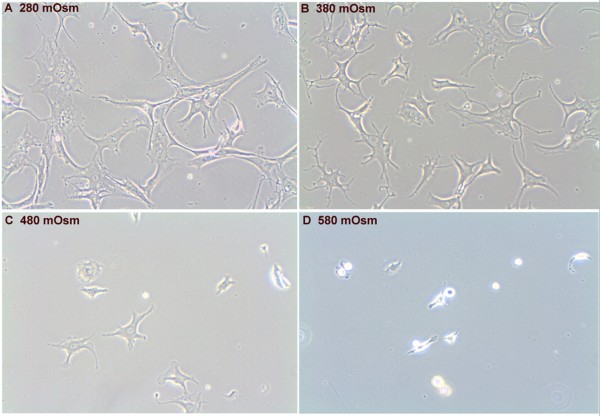
**Hypertonic isolation and expansion of chondrocytes changes chondrocyte morphology**. Representative images (200×) of chondrocytes cultured for 2 days at **(a) **280 mOsm, **(b) **380 mOsm, **(c) **480 mOsm and **(d) **580 mOsm.

We first determined the influence of tonicity on proliferation: OA HACs isolated at 580 mOsm hardly attached or proliferated (Figure 1d), and 2 days after seeding no viable cells were recovered. At 280 mOsm, 380 mOsm and 480 mOsm, respectively, cells did adhere but increasing tonicity induced marked morphological changes: at 280 mOsm, cells appeared fibroblast-like, stretched out and flattened with long filopodia (Figure 1a); while at 380 mOsm, cells were more sphere-shaped and had shorter filopodia (Figure 1b). At 480 mOsm, cells showed few filopodia and appeared spherical (Figure 1c). The differences in appearance remained throughout the dedifferentiation period (P0 to P3), but were most apparent at earlier passages.

Using cell counts and DNA assays, doubling times were calculated from growth curves established from each passage at three different tonicities (280 mOsm, 380 mOsm and 480 mOsm). Throughout dedifferentiation, OA HACs isolated at 480 mOsm showed severely inhibited proliferation compared with cells at 280 mOsm and 380 mOsm (Table 1). In contrast, doubling times of OA HACs at 280 mOsm and 380 mOsm never significantly differed (Table 1). All further experiments were therefore performed at 380 mOsm (as high tonicity condition) and compared with 280 mOsm (control condition).

**Table 1:** Proliferation of chondrocytes isolated and cultured at 280 mOsm, 380 mOsm and 480 mOsm

	Chondrocyte proliferation (%)			
	
Culture condition	Passage 0	Passage 1	Passage 2	Passage 3
280 mOsm	100 (68 ± 28 hours)	100 (89 ± 54 hours)	100 (67 ± 48 hours)	100 (57 ± 11 hours)
380 mOsm	113 ± 18	89 ± 25	99 ± 9	154 ± 41
480 mOsm	675 ± 405*	180 ± 24*	168 ± 28*	165 ± 81*

Data presented as relative doubling times in percentage of cells cultured at 280 mOsm, mean ± standard deviation. The absolute doubling time ± standard deviation in hours is displayed in brackets. n = 6. mOsm, milliosmoles per kilogram of water. *P < 0.05.

### Isolation and expansion of chondrocytes under hypertonic conditions improves their phenotype

**Figure 2:**
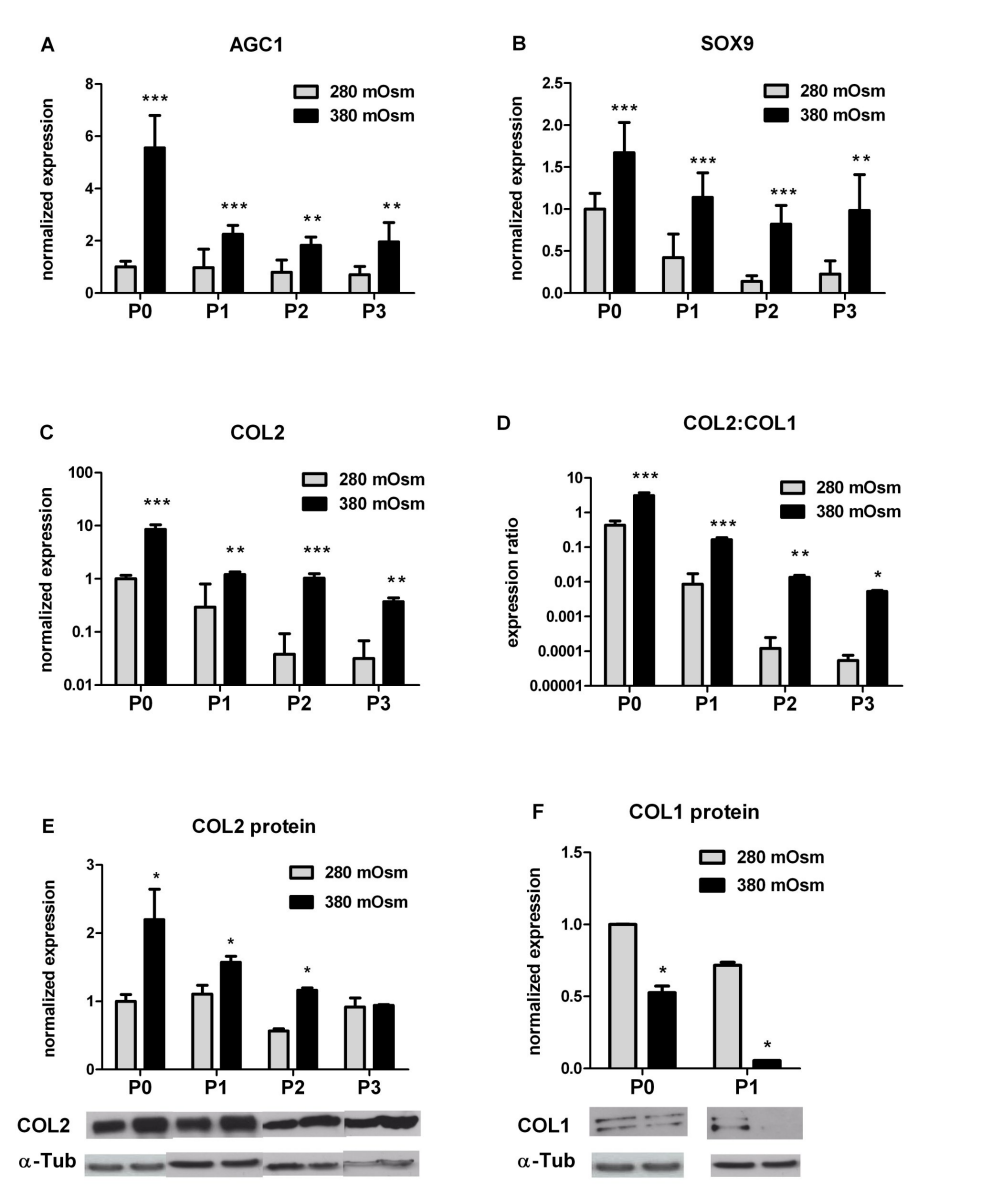
**Hypertonic isolation and expansion increased marker gene expression in osteoarthritis human articular chondrocytes**. Relative expression of **(a) ***AGC1*, **(b) ***SOX9*, **(c) ***COL2 *and **(d) ***COL2:COL1 *ratio in primary (P0) and passaged (P1 to P3) chondrocytes cultured at 380 mOsm compared with 280 mOsm. **(e) **COL2 protein expression and **(f) **COL1 protein expression in P0 and P1 osteoarthritis human articular chondrocytes. Protein levels normalized to α-tubulin. Data are mean ± standard deviation, n = 12. Differences from cells cultured at 280 mOsm are indicated: *P < 0.05, **P < 0.01 and ***P < 0.001.

Next, we set out to determine whether expansion culture in physiological tonicity improves the chondrocytic phenotype. Physiological tonicity (380 mOsm) during isolation and subsequent passaging of OA HACs significantly increased mRNA levels of both *AGC1 *(Figure 2a) and *SOX9 *(Figure 2b) at all passages. In expanded P3 chondrocytes in physiological culture, *AGC1 *levels were still higher than in unpassaged P0 chondrocytes cultured under the standard culture conditions (280 mOsm).

Physiological tonicity also significantly upregulated *COL2 *levels from 8.5-fold in P0 to 11.6-fold in expanded P3 chondrocytes (Figure 2c) compared with controls. In contrast, *COL1 *expression was significantly suppressed in physiological conditions throughout culture. Consequently, we found a significantly improved *COL2/COL1 *ratio during chondrocyte expansion (Figure 2d), from sevenfold in P0 cells to 100-fold in expanded P3 cells. Physiological tonicity also upregulated COL2 protein expression (Figure 2e): levels significantly increased (between 1.5-fold and 2.2-fold) in P0, P1 and P2 chondrocytes. In contrast, physiological tonicity significantly decreased COL1 protein expression (Figure 2f), from twofold in P0 cells to 13-fold in P1 cells.

**Figure 3:**
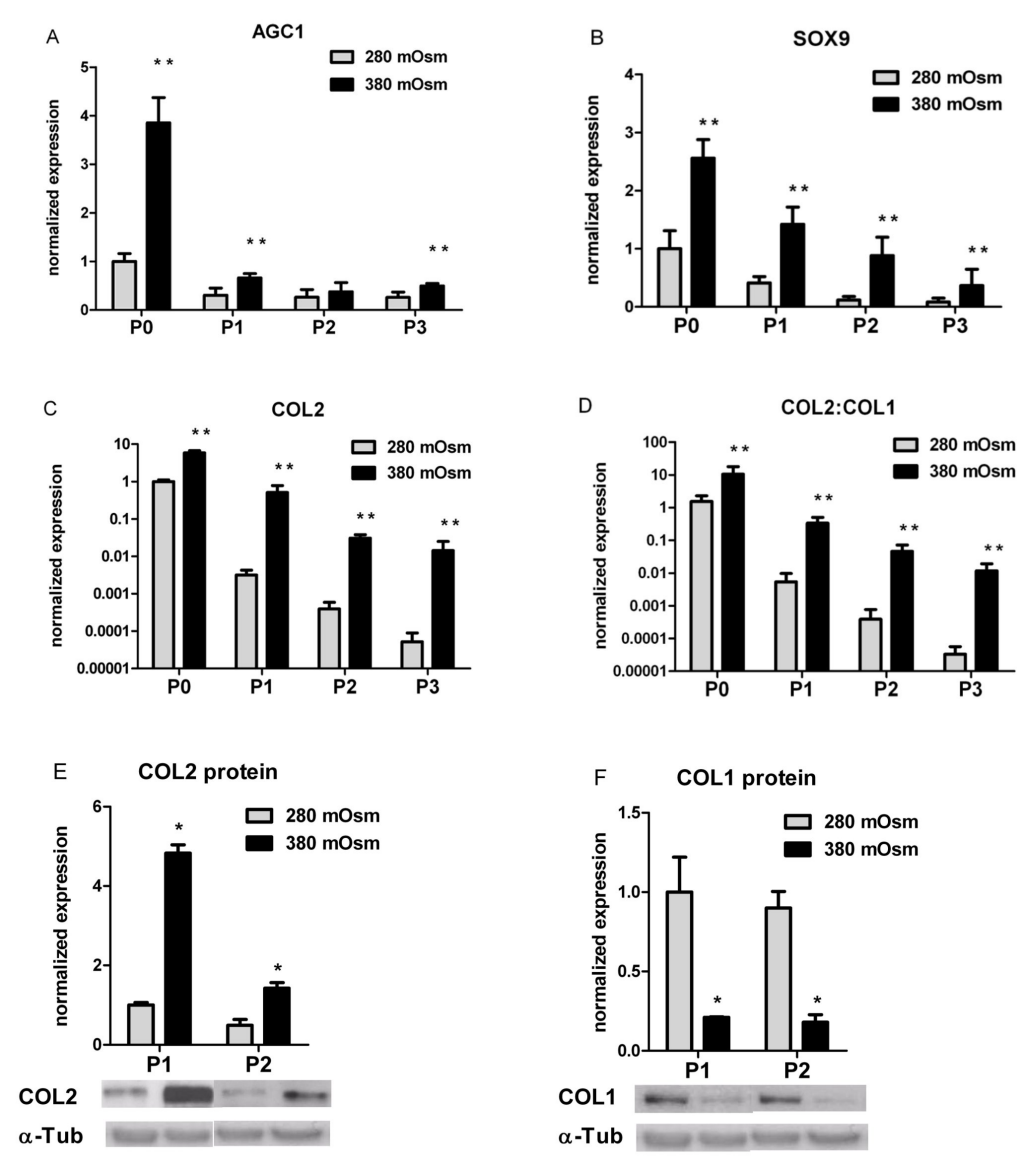
**Hypertonic isolation and expansion increased chondrogenic marker expression in nonosteoarthritic human articular chondrocytes**. Relative expression of **(a) ***AGC1*, **(b) ***SOX9*, **(c) ***COL2 *and **(d) ***COL2:COL1 *ratio in primary (P0) and passaged (P1 to P3) nonosteoarthritic human articular chondrocytes (NHACs) cultured at 380 mOsm compared with cells cultured at 280 mOsm. **(e) **COL2 protein expression and **(f) **COL1 protein expression in P1 and P2 NHACs, normalized to α-tubulin. Data are mean ± standard deviation, n = 6. Differences from 280 mOsm controls are indicated: *P < 0.05 and **P < 0.01.

Physiological tonicity also significantly increased *AGC1 *(Figure 3a) and *SOX9 *(Figure 3b) mRNA levels in nonosteoarthritic human articular chondrocytes (NHACs). Furthermore, *COL2 *mRNA levels were significantly upregulated, from 5.8-fold in P0 cells to 270-fold in expanded P3 NHACs (Figure 3c). As in OA HACs, hypertonicity also downregulated *COL1 *expression with increasing passage number in NHACs: the *COL2/COL1 *ratios increased during expansion (Figure 3d), from 6.8-fold in P0 cells to 355-fold in expanded P3 cells. Correspondingly, COL2 protein levels increased under these conditions (4.8-fold in P1 cells and 2.9-fold in P2 cells), while the amount of COL1 diminished (by 4.7-fold in P1 cells and fivefold in P2 cells) (Figure 3e, f).

### Hypertonicity activates *NFAT5* in human articular chondrocytes

**Figure 4:**
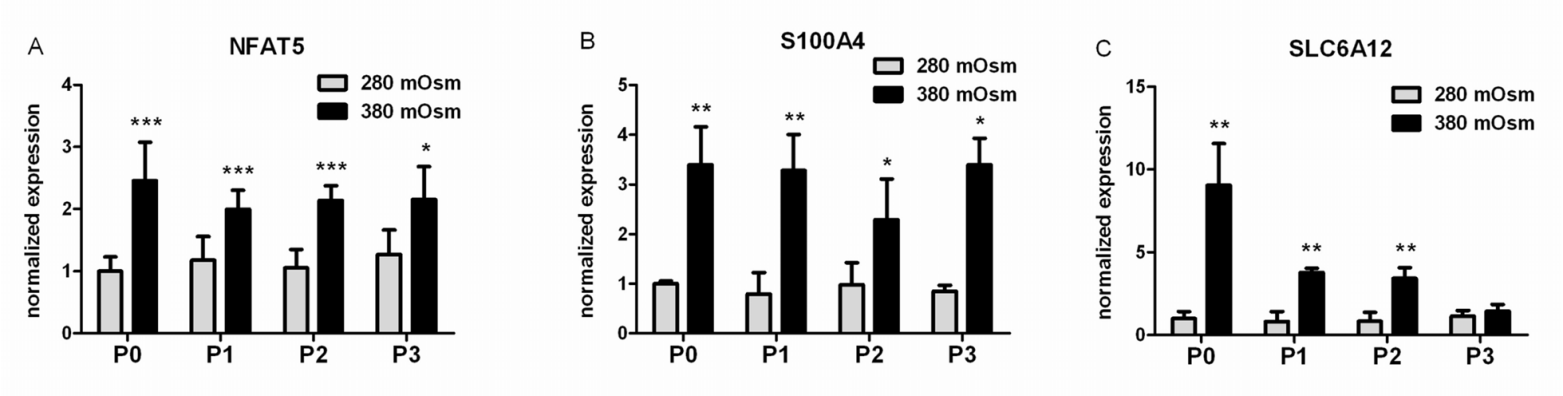
**Hypertonic conditions activate nuclear factor of activated T-cells 5 in osteoarthritis human articular chondrocytes**. Relative expression of **(a) **nuclear factor of activated T-cells 5 (*NFAT5*) and its target genes **(b) ***S100A4 *and **(c) **SLC6A12 in primary (P0) and passaged (P1 to P3) chondrocytes cultured at 380 mOsm compared with 280 mOsm. Data are mean ± standard deviation, n = 12. Differences are indicated: *P < 0.05, **P < 0.01 and ***P < 0.001.

Compared with 280 mOsm controls, *NFAT5 *mRNA levels were significantly increased in 380 mOsm OA HAC cultures (Figure 4a), as was the expression of established NFAT5 target genes *S100A4 *(in all passages; Figure 4b) and *SLC6A12 *(until P2; Figure 4c). Similar effects were found in NHACs (data not shown).

### *NFAT5* knockdown inhibits hypertonicity-induced chondrogenic marker expression

**Figure 5:**
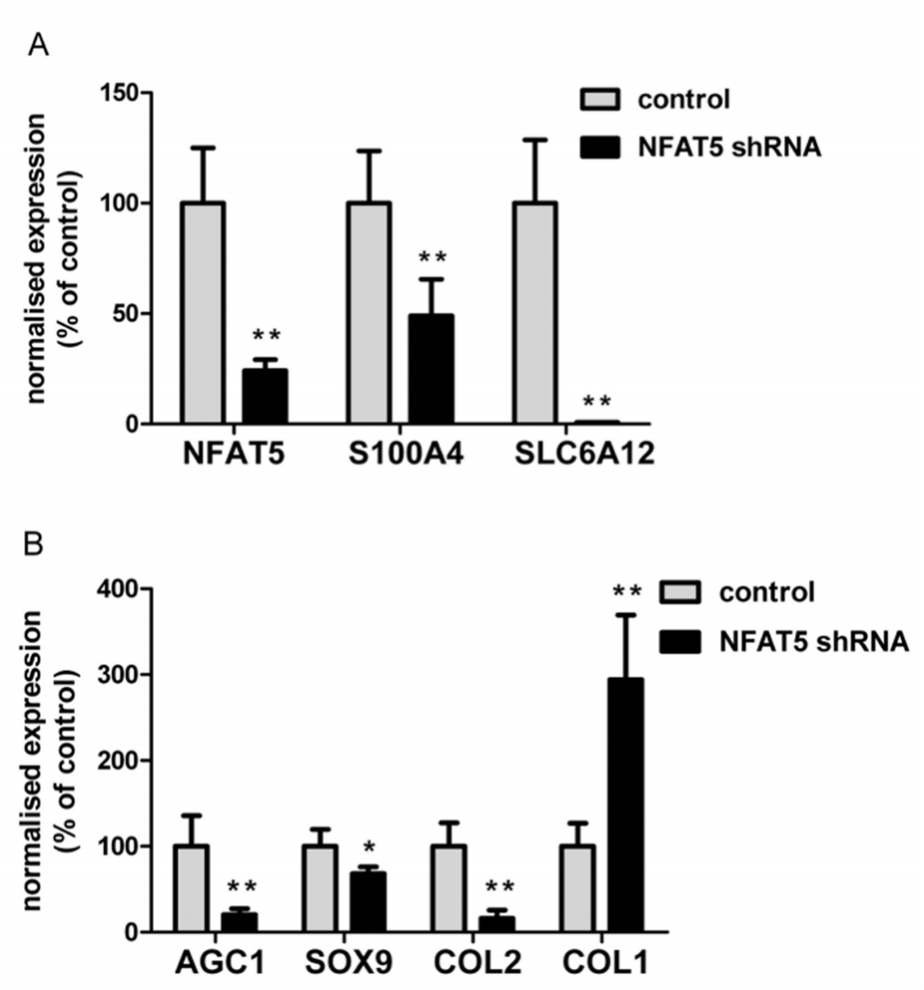
**Nuclear factor of activated T-cells 5 knockdown inhibits hypertonicity-induced chondrogenic marker expression**. **(a) **Relative expression of nuclear factor of activated T-cells 5 (*NFAT5*) and its target genes *S100A4 *and SLC6A12 in transduced chondrocytes either expressing (*NFAT5 *shRNA) or not expressing (control) *NFAT5*-specific shRNAs, 24 hours after increasing tonicity to 380 mOsm. **(b) **Effects of *NFAT5 *knockdown on chondrogenic markers *AGC1*, *SOX9*, *COL2 *and *COL1*. Data are mean ± standard deviation, n = 6. Differences from cells transduced with control virus are indicated: *P < 0.05 and **P < 0.01.

Upon transduction, sorted eGFP-coexpressing OA HACs were switched to 380 mOsm for 24 hours. In controls not expressing *NFAT5*-specific shRNAs, an approximately twofold increase in *NFAT5 *mRNA levels was observed upon hypertonic stimulation (Figure 4a, P1). In contrast, likewise challenged cells expressing anti-*NFAT5 *shRNAs showed an approximately 75% reduction in *NFAT5 *levels (Figure 5a). Following *NFAT5 *knockdown, the *NFAT5 *targets *S100A4 *and *SLC6A12 *were also no longer hypertonically inducible: *S100A4 *expression decreased twofold and *SLC6A12 *was virtually undetectable upon *NFAT5 *RNAi (Figure 5a), confirming a functional *NFAT5 *knockdown. At 380 mOsm, *NFAT5 *RNAi also downregulated chondrogenic markers: *AGC1 *by 80%, *SOX9 *by 32% and *COL2 *by 84%, as compared with non-RNAi controls (Figure 5b). Interestingly, expression of *COL1 *increased after *NFAT5 *RNAi in OA HACs to ~300% of control levels (Figure 5b).

## Discussion

Isolation and expansion of adult HACs under physiological tonicity (380 mOsm) improves expression of chondrogenic markers on mRNA and protein levels. While other studies partially confirm that nonhuman chondrocytes respond to tonicity with altered aggrecan and *SOX9 *expression [4,8,10], we are reporting beneficial effects of isolating and expanding human normal and OA articular chondrocytes at physiological levels (380 mOsm). In addition, we also studied collagen type II expression, generally acknowledged to be the most important chondrogenic marker. As fibrocartilaginous collagen type I and hyaline collagen type II expression are differentially regulated in chondrocytes [34], analyzing the collagen type II/type I expression ratios is informative of chondrogenic potential [51]. Interestingly, NFAT5 seems to be crucially involved in this differential regulation upon hypertonic challenge: it positively regulates collagen type II, while suppressing collagen type I (Figure 5b). Fibrocartilage, occurring in areas subject to frequent stress like intervertebral discs and tendon attachment sites, is more rich in collagen type I than is hyaline cartilage [55]. Tonicity may thus provide a simple means to manipulate expression of these two collagens for broader applications than regenerative chondrocyte implantations (autologous chondrocyte implantation or characterized chondrocyte implantation) alone [56].

Under our conditions, *COL2 *mRNA abundances measured by quantitative PCR correlated well with protein synthesis as determined by Western blots (Figures 2 and 3). The same observation holds for *COL1 *expression in the early passages, but not for *COL1 *expression in the later passages.

Hypertonicity induced an increase in NFAT5 abundance, and protein synthesis rates were found to be proportional to the increase in mRNA in MDCK cells [28] and mIMCD3 cells [27]. *NFAT5 *mRNA is expressed abundantly in chondrocytes throughout passages and is further induced by hypertonicity. However, we failed to show NFAT5 protein expression by Western blotting. Whether this failure is due to low protein abundance in our cells or technical issues such as poor extraction efficiency of this very large transcription factor remains to be elucidated in future experiments.

Hypertonicity induces cell shrinkage, which may activate Na^+^, K^+^, or 2Cl^- ^co-transport, allowing cellular accumulation of NaCl and KCl. The beneficial effects on chondrogenic marker gene expression therefore could have been caused by accumulation of specific inorganic ions or specific channel activity rather than primarily tonicity-mediated effects. We used NMDG-Cl, a bulky substitute for small cations that is impermeable to almost all known channels [57], and sucrose to exclude sodium-specific or chloride-specific effects. We were not able to detect any significant differences in gene expression patterns between the NaCl, NMDG-Cl or sucrose methods of tonicity alteration (data not shown).

As our initial studies concerned adult HACs obtained from OA knee joints, we aimed at eliminating interpretation bias due to the pathological state of these cells. Using identically challenged NHACs, we showed that these chondrocytes react similarly to the same order of tonicity with respect to our marker genes: 380 mOsm significantly delayed the phenotypical deterioration of NHACs as observed in control medium. This may imply that physiological tonicity, postulated to be around 380 mOsm for chondrocytes, is sensed by OA cells and normal cells in a similar fashion. We observed a slightly faster decrease in *AGC1 *and *COL2 *mRNA levels in P2 and P3 NHACs as compared with OA HACs. Late-stage OA chondrocytes from fibrillated areas are dedifferentiated, flattened cells. The loss of a proper spherical shape as an integral part of the chondrocytes phenotype [58,59] involves cytoskeletal changes [60]. Exposing these cells to physiological tonicity as a redifferentiation stimulus probably induces a more enduring response as compared with spherical, normal chondrocytes. Cell-based therapies using the latter are usually restricted to younger individuals after traumatic insults. Autologous chondrocyte implantation employing OA cells may benefit relatively more from a hypertonic treatment protocol.

The precise molecular mechanism by which tonicity is sensed by cells is still poorly understood. Hypertonicity-increased *NFAT5 *mRNA abundances have been shown for other cell types [26-28]. NFAT5 is thus accepted as key transcription factor participating in the mammalian hypertonic stress response. Our study is the first showing the functional expression of *NFAT5 *in HACs. In both OA and normal chondrocytes, cellular *NFAT5 *mRNA levels are increased by 380 mOsm. In addition, mRNA levels of the generally accepted NFAT5 target genes, *S100A4 *and *SLC6A12 *[20,61], were induced accordingly after hypertonic challenge, underscoring an involvement of *NFAT5*. It has recently been suggested that guanine nucleotide exchange factors near the plasma membrane may be activated through cytoskeleton changes or by changes in interactions with putative osmosensors at the cell membrane in other cells [62]. The sensation of such basic responses might not be different in chondrocytes than in other cells. Rho-type small G proteins [63] and p38 kinases [64,65] might also act upstream of NFAT5 in chondrocytes. In IMCD cells, p38 mitogen-activated protein kinase (MAPK) signaling was recently also shown to be involved in the *NFAT5*-mediated hypertonic induction of the osmosensitive [66,67] serine-threonine protein kinase Sgk-1 [68,69]. As p38 MAPK plays important roles in chondrocytes and seems to be necessary for *NFAT5 *expression [20], further experiments employing pharmacological inhibition or knockdown experiments in HACs will hopefully shed more light into this signaling cascade in chondrocytes.

An increase in NFAT5 mRNA is usually transient with a cell type-dependent time course and a twofold to fourfold upregulation [26,28], which fits with our data. *NFAT5 *mRNA abundance might rapidly increase upon hypertonic stress by a transient increase in its mRNA stability, mediated by its 5'-untranslated region [27]. Whether 380 mOsm is a sufficiently high tonicity to explain our increase in mRNA by this phenomenon, or whether active transcription is involved, has to be addressed in other studies. Interestingly, Tew and colleagues showed very recently that the mRNA of *SOX9*, an important regulator of COL2 expression, is stabilized by supraphysiological tonicity [70]. Therefore, 380 mOsm might also directly contribute to *SOX9 *mRNA stability and abundance in our experiment, rather than elevating promoter activity. COL2 regulation could thus be an indirect effect of tonicity.

Interestingly, AGC1 seems to be more stably expressed in cultures maintained at 280 mOsm compared with 380 mOsm, with a lower overall expression in the former condition. Effects of tonicity on promoter activity and mRNA stability of *AGC1 *are incompletely understood. Other groups have described the complexity of osmotic stress on gene expression [71,72]. It is tempting to speculate that gene expression may be influenced by morphological changes between our conditions: while cells cultured at 380 mOsm are rather round, cells cultured in monolayer at 280 mOsm are rather flat and more fibroblast-like (see Figure 1). Although we did not investigate actin stress fiber formation in the present study, they are usually more pronounced in fibroblastic cells and have been shown to suppress *SOX9 *mRNA levels in chondrocytes [50].

Aggrecan expression, however, has been reported to be influenced by both hypertonicity and hypotonicity [4,8]. The promoter regions of both collagen type II and *AGC1 *contain a plethora of potential other binding sites for transcriptional enhancers and suppressors, such as SOX5/6 [73,74], Barx2 [75], β-catenin [76], c-Maf [77], PIAS [78], TRAP230 [79], Bapx1 [80], and C/EBP and NF-κB [81]. Chondrogenic differentiation and the SOX9 dependency of aggrecan and collagen expression may also be differentially modulated by these transcriptional cofactors under different tonicities. Interestingly, while the SOX9 dependency of COL2A1 expression has been unequivocally shown, it may not actually be a key regulator of *COL2A1 *promoter activity in human adult articular chondrocytes [82]. Of note, the human aggrecan promoter sequence has been shown to contain a conserved NFAT5 binding site [83]. In nucleus pulposus cells, SOX9-mediated aggrecan expression has recently been shown to critically depend on PI3K/AKT signaling [84]. Moreover, while high NaCl rapidly activates p38 MAPK, its action can be isoform specific and may exert opposing effects on NFAT5 [85], which in turn may influence *COL2A1 *and *AGC1 *transcription differently in a tonicity-dependent manner. We are therefore currently looking into the underlying molecular mechanisms regulating AGC1 and COL2 expression in both conditions.

With respect to regenerative medical applications, the high-end hypertonic conditions used by Tew and colleagues can be considered a limitation of that study. In our hands, these tonicity levels (≥ 480 mOsm) induced chondrocyte death within 48 hours (Figure 1d) and are probably not applicable for chondrocyte expansion culture. To ensure sufficient cell numbers for cell-based repair techniques, the proliferation capacity of the isolated chondrocytes should not be compromised. Cell numbers generally need to be increased during two passages (>4 to 10 times) for clinical application [86,87]. We found that supraphysiological conditions (480 mOsm and 580 mOsm) clearly compromised survival rates, which is in agreement with data by Racz and colleagues [17]. From our data, we conclude that about 380 mOsm is optimal for both isolation and *in vitro *expansion culture of HACs.

NFAT5 knockdown downregulates its own transcription by 75% and compromises target gene induction (Figure 5), being in line with functionally active NFAT5 in chondrocytes. Constitutive homodimeric NFAT5 molecules encircle DNA rather independently of tonicity in solution [88], enabling NFAT5 to exert its biological activity over a wide tonicity range [89,90]. It is thus reasonable to assume that NFAT5 activity is not generally compromised at 380 mOsm. However, other aspects are involved in the regulation of NFAT5 as well as its target genes. Like other proteins larger than 50 kDa [91], NFAT5 depends on nuclear localization and export sequences for its nuclear translocation [26,88,91]. In most cells, NFAT5 is equally distributed between the cytoplasm and the nucleus at physiological tonicity (± 300 mOsm), whereas at 500 mOsm most of it localizes to the nucleus [19,26,89].

To demonstrate that the hypertonicity-induced chondrogenic marker expression was indeed mediated by NFAT5, we used RNAi to confirm that knockdown of NFAT5 significantly inhibited hypertonic induction of its own transcription as discussed before, significantly suppressed the tonicity-mediated induction of known NFAT5 targets, and, most importantly, significantly eliminated the hypertonicity-mediated mRNA expression of chondrogenic marker genes (*COL2*, *AGC1*, *SOX9 *and *COL1*).

## Conclusions

We have shown that isolation and expansion of adult HACs in culture medium of physiological tonicity (380 mOsm) improves chondrogenic marker expression and extracellular matrix production through NFAT5. We identified NFAT5 as a novel molecular target preserving chondrocytic marker expression. Our data provide valuable insights for the development of strategies for cell-based repair of chondral lesions, and contribute to the understanding of mechanisms involving OA.

## Abbreviations

DMEM: Dulbecco's modified Eagle's medium; eGFP: enhanced green fluorescent protein; FACS: fluorescence-activated cell sorting; FCS: fetal calf serum; HAC: human articular chondrocyte; MAPK: mitogen-activated protein kinase; mOsm: milliosmoles per kilogram of water; NF: nuclear factor; NFAT: nuclear factor of activated T cells; NHAC: nonosteoarthritic human articular chondrocyte; NMDG-Cl: *N*-methyl-d-glucamine chloride; OA: osteoarthritis; PBS: phosphate-buffered saline; PCR: polymerase chain reaction; P: passage; RNAi: RNA interference; RT: reverse transcriptase; TonEBP: Tonicity-responsive Enhancer Binding Protein.

## Competing interests

The authors declare that they have no competing interests.

## Authors' contributions

HJ conceived the study. AEvdW, HW, JANV and HJ designed the study. AEvdW, EH and RHJD analyzed the data. AEvdW, EH, NK, TJMW and MMJC performed the experiments. NPvT, TJMW and MMJC contributed the reagents/materials/analysis tools. AEvdW and HJ wrote the paper. All authors read and approved the final manuscript.
